# Patterns, Clinical Management, and Short-Term Outcomes of Acute Limb Ischemia (ALI) Seen In In-Hospital and Out-of-Hospital Onset

**DOI:** 10.7759/cureus.101475

**Published:** 2026-01-13

**Authors:** Kayoko Natsume, Nene Imai, Maiko Yoshida, Keita Hosaka, Hiroshi Nagano, Syunsuke Miyahara, Mitsuru Asano

**Affiliations:** 1 Division of Cardiovascular Surgery, Seirei Mikatahara General Hospital, Hamamatsu, JPN; 2 Division of Vascular Surgery, Hamamatsu University School of Medicine, Hamamatsu, JPN

**Keywords:** acute limb ischemia, in-hospital onset, malignancy, thrombectomy, vascular surgery

## Abstract

Background

Acute limb ischemia (ALI) is a vascular emergency associated with high morbidity and mortality. While most studies focus on community-onset ALI, some patients develop ALI during hospitalization for other medical conditions, where outcomes and decision-making may differ. Data on the in-hospital onset ALI remain limited.

Aims

This study aimed to evaluate the clinical characteristics and outcomes of patients with in-hospital onset ALI compared with those with out-of-hospital onset at a single center.

Methods

We retrospectively reviewed patients who underwent emergency surgical thrombectomy for ALI at a single center between April 2019 and August 2025. Patients with vascular access-related ALI, iatrogenic ALI, or bypass graft occlusion were excluded. ALI was classified as an in-hospital or out-of-hospital onset. Outcomes included 30-day mortality, in-hospital mortality, and major amputation.

Results

Twenty-five limbs in 25 patients were analyzed (right: 18, left: seven): 19 with out-of-hospital onset and six with in-hospital onset ALI. The in-hospital onset group had a higher prevalence of active cancer and infection. Operative variables and time from onset to revascularization were similar between groups. No major amputations were performed. However, one in-hospital onset patient developed irreversible dry gangrene and died from terminal cancer. Thirty-day mortality was significantly higher in the in-hospital onset group (50% vs. 0%).

Conclusion

In-hospital onset ALI is associated with markedly higher early mortality despite comparable limb-related outcomes. Prognosis appears to be driven primarily by systemic conditions rather than limb ischemia alone. Although in-hospital onset ALI represents a high-risk subgroup, outcomes are not uniformly fatal, underscoring the importance of individualized treatment decisions based on overall clinical status.

## Introduction

Acute limb ischemia (ALI) is a vascular emergency that can be both life- and limb-threatening. Despite advances in revascularization strategies, ALI remains associated with substantial short-term mortality and amputation rates [1-3]. The true incidence of ALI is difficult to determine owing to heterogeneous clinical presentations and treatment pathways; however, previous reports have estimated the incidence to be approximately nine to 16 cases per 100,000 persons per year [4-6].

Outcomes of ALI are influenced not only by limb-related factors but also by patients’ systemic conditions. In particular, ALI occurring in the context of serious underlying diseases such as malignancy, infection, or advanced organ dysfunction poses additional challenges in clinical decision-making. Several studies have reported wide-ranging short-term mortality and amputation rates in patients with ALI and underlying malignancy, reflecting the wide variability in patient backgrounds and systemic disease burden [7,8].

Most previous studies have focused on patients who develop ALI outside the hospital. In contrast, a distinct subgroup of patients develops ALI while hospitalized for other medical conditions. In these cases, conventional timelines used to evaluate treatment delay, such as symptom onset, hospital arrival, and time to surgery, may not be directly applicable. Furthermore, treatment decisions are often complicated by severe systemic deterioration, competing therapeutic priorities, and uncertainty regarding overall prognosis. A potential advantage of in-hospital onset ALI is immediate access to medical care, which may facilitate earlier diagnosis and revascularization [9]. However, patients who develop ALI during hospitalization are often treated for other serious conditions, and severe systemic illness may outweigh this potential benefit. Although in-hospital onset ALI is frequently perceived in clinical practice as being associated with poorer outcomes, data specifically addressing this subgroup remain limited [4].

Given these considerations, the present study aimed to evaluate the clinical characteristics and outcomes of patients with in-hospital onset ALI compared with those with out-of-hospital onset at a single center.

## Materials and methods

Study design

This was a single-center retrospective study conducted at Seirei Mikatahara Hospital in Hamamatsu, Japan.

Study population

A total of 37 patients underwent emergency surgical thrombectomy for ALI between April 2019 and August 2025. Patients with vascular access-related ALI, iatrogenic (procedure-related) ALI, or bypass graft occlusion were excluded (n = 12). Finally, 25 patients (25 limbs) (right: 18, left: 7) were included in the analysis.

Definitions and data collection

ALI cases were classified according to the onset setting. “In-hospital onset” was defined as ALI occurring in patients who were already under active medical care for other diseases at the time of onset, regardless of whether they were hospitalized at our institution or another medical institution. This definition was intended to distinguish patients who developed ALI while already under physician supervision from those who developed ALI while living independently in the community and presenting from home, defined as “out-of-hospital onset.” Accordingly, this classification reflects differences in clinical context and baseline systemic condition rather than physical location alone.

Of these, 19 patients had out-of-hospital onset ALI and six had in-hospital onset ALI. “Acute on chronic” was defined as the presence of chronic ischemic symptoms, collateral circulation, or imaging findings suggesting pre-existing arterial disease [10]. ALI severity at presentation was graded using the Rutherford classification, which categorizes limbs as Class I (viable), Class IIa/IIb (marginally to immediately threatened), or Class III (irreversible ischemia) based on clinical findings of limb viability [1].

Outcomes

The outcomes of interest were 30-day mortality, in-hospital mortality, and major amputation.

Statistical analysis

Statistical analyses were performed using IBM SPSS Statistics software (version 22, IBM Corp., Armonk, NY, USA). Categorical variables were presented as frequencies and percentages and were compared using Fisher’s exact test. Continuous variables were presented as median (interquartile range) and were compared using the Mann-Whitney U test. Statistical significance was defined as p < 0.05.

Ethical approval

This study was approved by the institutional review board of Seirei Mikatahara Hospital (approval number: 24-39). The requirement for informed consent was waived due to the retrospective nature of the study. 

## Results

A total of 25 patients (25 limbs) were analyzed: 19 with out-of-hospital onset and six with in-hospital onset ALI. Baseline characteristics are shown in Table 1.

**Table 1:** Baseline characteristics Categorical variables were compared using Fisher’s exact test, and continuous variables were compared using the Mann–Whitney U test. p < 0.05 was considered statistically significant.

Variable	Out-of-hospital onset (n=19)	In-hospital onset (n=6)	Test statistic	p-value
Age, median (min-max)	80 (50–98)	80 (67–87)	U=53.5	0.914
Sex, male (%)	12 (63%)	5 (83%)	-	0.349
Hypertension	15 (79%)	4 (67%)	-	0.729
Diabetes mellitus	5 (26%)	3 (50%)	-	0.274
Hyperlipidemia	12 (63%)	3 (50%)	-	0.455
Cerebrovascular disease	2 (11%)	2 (33%)	-	0.234
Atrial fibrillation	14 (74%)	2 (33%)	-	0.097
Cancer	0 (0%)	3 (50%)	-	0.009*
Infection	0 (0%)	3 (50%)	-	0.009*
Antiplatelets	2 (11%)	0 (0%)	-	0.57
Anticoagulation drugs	1 (5%)	2 (33%)	-	0.579

The in-hospital onset group more frequently had active malignancy and infection, whereas cardiovascular comorbidities such as atrial fibrillation and hypertension were comparably distributed between groups.

Operative variables showed no significant differences between the two groups (Table 2), including operative time, anesthesia type, Rutherford classification, and distribution of target vessels. There was also no significant difference in the time from onset to operation. In the out-of-hospital onset group, five patients underwent additional surgical revascularization (bypass or endarterectomy with patch plasty) after thrombectomy because of residual stenosis, despite successful initial thrombectomy in all patients.

**Table 2:** Operative findings Categorical variables were compared using Fisher’s exact test, and continuous variables were compared using the Mann–Whitney U test. p < 0.05 was considered statistically significant. Creatine kinase was measured as a surrogate marker of muscle injury.

Variable	Out-of-hospital onset (n=19)	In-hospital onset (n=6)	Test statistic	p-value
Time from onset (days)	1.0 (0.2-15)	0.3 (0.1-1.0)	U=76.0	0.223
Rutherford class 1/2a/2b	6 (32%) /9 (47%) /4 (21%)	2 (33%) /3 (50%) /1 (17%)	-	0.973
Preoperative creatine kinase (U/L)	255 (31–89852)	124 (25–2049)	U=47.0	0.641
Acute on chronic	7 (37%)	1 (17%)	-	0.349
Operative findings				
Operation time (minutes)	92 (32–218)	97 (57–229)	U=63.0	0.703
General anesthesia	8 (42%)	1 (17%)	-	0.267
Surgical thrombectomy	19 (100%)	6 (100%)	-	
Iliac artery	2 (11%)	2 (30%)	-	
Femoropopliteal artery	15 (79%)	6 (100%)	-	
Infrapopliteal artery	1 (5%)	0 (0%)	-	
Upper limb artery	3 (16%)	0 (0%)	-	
Failed thrombectomy	0 (0%)	1 (17%)	-	0.24
Endovascular revascularization	0 (0%)	0 (0%)	-	
Surgical revascularization	5 (26%)	0 (0%)	-	0.289

No major amputations were performed in either group. In the in-hospital onset group, one patient progressed to irreversible dry gangrene. In this patient with terminal cancer, a major amputation was not performed because the cause of death was attributed to cancer progression rather than ALI. Postoperative mortality differed significantly between the groups (Table 3).

**Table 3:** Postoperative outcomes Categorical variables were compared using the Fisher’s exact test. p < 0.05 was considered statistically significant.

Variable	Out-of-hospital onset (n=19)	In-hospital onset (n=6)	Test statistics	p-value
Fasciotomy	1 (5%)	0 (0%)	-	1
30-day mortality	0 (0%)	3 (50%)	-	0.009*
In-hospital mortality	0 (0%)	3 (50%)	-	0.009*
30-day major amputation	0 (0%)	0 (0%)	-	

The 30-day and in-hospital mortality rates were 50% (3/6) in the in-hospital onset group, whereas no deaths occurred in the out-of-hospital onset group (p = 0.009), with deaths primarily related to systemic deterioration rather than limb-related complications.

Detailed clinical information for in-hospital onset cases is shown in Table 4.

**Table 4:** Detailed clinical characteristics of patients with in-hospital onset ALI ALI: acute limb ischemia; Af: atrial fibrillation; POD: postoperative day; POM: postoperative month; MOF: multiple organ failure; BSC: best supportive care

Case	Sex	Age (years)	Reason for admission	Rutherford classification	Time from onset to operation (hours)	Time from admission to onset (days)	Af	Anticoagulation	Cause of ALI	Procedure	Outcome
1	Female	87	Rectal cancer/ileus	2a	6	2	+	-	Embolism	Thrombectomy	Survived, ambulatory
2	Male	67	Infection (unknown source)/dehydration	2a	12	1	-	-	In-situ thrombosis	Thrombectomy	Survived, ambulatory
3	Male	87	Pneumonia, GI bleeding	2b	6	20	+	-	Embolism	Thrombectomy	Died POD1 (MOF)
4	Male	82	Pharyngeal cancer with multiple metastases	1	24	9	-	-	Acute on chronic	Thrombectomy (failed)	Died POM1 (BSC)
5	Male	68	Ureteral cancer surgery	1	3	1	-	-	Embolism	Thrombectomy	Survived, ambulatory
6	Male	79	Cerebral infarction (paradoxical embolism)/aspiration pneumonia	2a	4	46	-	+	Embolism	Thrombectomy	Died POM1 (Pneumonia)

Among the six patients, three had active malignancy, and three had systemic infection, while atrial fibrillation was present in only one patient. No major amputations were performed. Three patients died due to systemic deterioration rather than limb-related complications.

## Discussion

The major finding of this study is that in-hospital onset of ALI was associated with significantly higher mortality, despite comparable limb-related outcomes between groups. Although no major amputations were performed in either group, one patient in the in-hospital onset group developed dry gangrene and subsequently died due to progression of terminal cancer. Thus, the absence of major amputation did not necessarily indicate successful limb salvage in this case.

Previous studies have reported poor outcomes in ALI, with short-term mortality rates ranging from 11.4% to 28.9% and amputation rates of 14.3% to 30% [1-3].

Outcomes in patients with ALI associated with malignancy remain poor, with reported short-term mortality rates ranging from 3.5% to 50% and amputation rates of 4.2% to 37.5% [7,8,11-15]. In the present study, the overall 30-day mortality rate was 20%. However, mortality differed markedly according to the onset setting. No deaths occurred in patients with out-of-hospital onset ALI, whereas the 30-day mortality rate reached 50% in patients with in-hospital onset ALI, despite comparable operative variables and the absence of major amputations. Three of six patients in our study died due to systemic deterioration, including one case in which thrombectomy failed and dry gangrene developed. These findings indicate that systemic condition rather than limb status was the dominant determinant of prognosis.

Time from onset to revascularization did not differ significantly between groups, suggesting that delayed intervention alone was not the primary reason for poor outcomes. However, decision-making in these patients can be challenging; in one case, surgery was deferred for more than 24 hours because of severe systemic deterioration, and the patient was initially managed with anticoagulation. Progressive ischemic symptoms necessitated thrombectomy, highlighting the difficulty in balancing systemic optimization and timely revascularization in critically ill patients.

This complexity is not unique to our cohort. In a population-based study from Gloucestershire, early mortality remained high even when both conservatively and actively treated patients were considered together, with 19 deaths among 80 hospital-managed events (approximately 24%). Notably, a distinct subgroup of patients who developed ALI while hospitalized for other medical conditions had particularly poor outcomes, with all nine patients dying despite selected attempts at active treatment [4].

It should be noted that this population-based study was conducted more than two decades ago, reflecting a different era of perioperative care, patient selection, and supportive management. In contrast, although in-hospital onset ALI in our cohort remained associated with high mortality, half of these patients survived following revascularization.

These findings suggest that while in-hospital onset ALI represents a particularly high-risk subgroup, outcomes may not be uniformly fatal in contemporary practice. Therefore, treatment decisions should not be based on onset setting alone.

Taken together, these findings suggest that although in-hospital onset ALI represents a particularly high-risk subgroup, outcomes are not uniformly fatal in contemporary practice. Historical population-based data reported extremely poor outcomes in this setting; however, more recent evidence indicates that selected patients, including those with underlying malignancy, may still benefit from active revascularization. In line with this evolving evidence base, the 2020 European Society for Vascular Surgery clinical practice guidelines on acute limb ischemia recommend that active revascularization should be considered in selected patients with ALI and underlying malignant disease (Class IIa, Level B), highlighting the importance of individualized decision-making. In our cohort, some cancer patients survived with good limb function following revascularization, suggesting that cancer alone should not preclude surgical intervention [16]. Treatment decisions should carefully consider systemic disease burden, prognosis, and patient-centered goals.

Limitations

This study has several limitations. The small sample size and retrospective, single-center design limit generalizability. Additionally, long-term functional outcomes were not evaluated, and selection bias may have influenced treatment decisions. Moreover, this analysis was limited to patients who underwent surgical thrombectomy; patients with ALI who were not referred to vascular surgeons or were deemed unsuitable for surgical intervention were not captured, which may have led to an underestimation of the true mortality in in-hospital onset cases. Further prospective studies with larger cohorts are warranted to clarify prognostic factors in this high-risk population.

## Conclusions

In-hospital onset ALI was associated with significantly higher early mortality compared with out-of-hospital onset cases. Although no major amputations were performed, survival was mainly limited by systemic conditions rather than limb-related factors. Importantly, however, not all patients with in-hospital onset ALI had uniformly poor outcomes. Selected patients achieved favorable survival and limb function following revascularization.

These findings underscore that in-hospital onset ALI represents a high-risk but heterogeneous clinical entity. Therefore, treatment decisions should neither be uniformly aggressive nor uniformly conservative, but rather individualized based on overall clinical status, systemic disease burden, and patient-centered goals.

## References

[1] Norgren L, Hiatt WR, Dormandy JA, Nehler MR, Harris KA, Fowkes FG (2007). Inter-Society consensus for the management of peripheral arterial disease (TASC II). J Vasc Surg.

[2] Fagundes C, Fuchs FD, Fagundes A, Poerschke RA, Vacaro MZ (2005). Prognostic factors for amputation or death in patients submitted to vascular surgery for acute limb ischemia. Vasc Health Risk Manag.

[3] Eliason JL, Wainess RM, Proctor MC (2003). A national and single institutional experience in the contemporary treatment of acute lower extremity ischemia. Ann Surg.

[4] Davies B, Braithwaite BD, Birch PA, Poskitt KR, Heather BP, Earnshaw JJ (1997). Acute leg ischaemia in Gloucestershire. Br J Surg.

[5] Dryjski M, Swedenborg J (1984). Acute ischemia of the extremities in a metropolitan area during one year. J Cardiovasc Surg (Torino).

[6] Creager MA, Kaufman JA, Conte MS (2012). Clinical practice. Acute limb ischemia. N Engl J Med.

[7] Mouhayar E, Tayar J, Fasulo M (2014). Outcome of acute limb ischemia in cancer patients. Vasc Med.

[8] Kelly A, Toale C, Moloney MA, Kavanagh EG (2022). Outcomes of acute limb ischaemia in patients with underlying malignancy: a systematic review. EJVES Vasc Forum.

[9] Rosenberg H, Rosenberg E, Kubelik D (2023). Acute limb ischemia. CMAJ.

[10] Doita T, Kikuchi S, Tamaru Y (2025). Clinical features of acute on chronic lower limb ischemia and the importance of underlying arterial disease for revascularization. Circ Rep.

[11] Javid M, Magee TR, Galland RB (2008). Arterial thrombosis associated with malignant disease. Eur J Vasc Endovasc Surg.

[12] Morris-Stiff G, Lewis MH (2010). Surgical treatment of acute limb ischaemia in the presence of malignancy. Int J Surg.

[13] Tsang JS, Naughton PA, O'Donnell J, Wang TT, Moneley DS, Kelly CJ, Leahy AL (2011). Acute limb ischemia in cancer patients: should we surgically intervene?. Ann Vasc Surg.

[14] Silverberg D, Yalon T, Reinitz ER, Yakubovitch D, Segev T, Halak M (2015). Acute limb ischemia in cancer patients: aggressive treatment is justified. Vascular.

[15] Bennett KM, Scarborough JE, Shortell CK, Cox MW (2014). Outcomes of surgical revascularization for lower extremity arterial thromboembolism in patients with advanced malignancy. J Vasc Surg.

[16] Björck M, Earnshaw JJ, Acosta S (2020). Editor’s choice - European Society for Vascular Surgery (ESVS) 2020 clinical practice guidelines on the management of acute limb ischaemia. Eur J Vasc Endovasc Surg.

